# Could protein content of Urinary Extracellular Vesicles be useful to detect Cirrhosis in Alcoholic Liver Disease?

**DOI:** 10.7150/ijbs.59725

**Published:** 2021-05-05

**Authors:** Esperanza Gonzalez, Mikel Azkargorta, Clara Garcia-Vallicrosa, Janire Prieto-Elordui, Felix Elortza, Sonia Blanco-Sampascual, Juan Manuel Falcon-Perez

**Affiliations:** 1 Exosomes Laboratory. Center for Cooperative Research in Biosciences (CIC bioGUNE), Basque Research and Technology Alliance (BRTA), Derio, Spain.; 2 Proteomics Platform. Center for Cooperative Research in Biosciences (CIC bioGUNE), Basque Research and Technology Alliance (BRTA), Derio, Spain.; 3 Servicio de Aparato Digestivo, Hospital Universitario de Basurto, Bilbao, Spain.; 4 Centro de Investigación Biomédica en Red de enfermedades hepáticas y digestivas (CIBERehd), Madrid, Spain.; 5 IKERBASQUE Basque Foundation for Science Bilbao Spain.

**Keywords:** urinary extracellular vesicles (uEVs), biomarkers, liquid biopsy, cirrhosis, fibrosis, alcoholic liver disease (ALD)

## Abstract

Alcohol abuse has a high impact on the mortality and morbidity related to a great number of diseases and is responsible for the development of alcoholic liver disease (ALD). It remains challenging to detect and evaluate its severity, which is crucial for prognosis. In this work, we studied if urinary EVs (uEVs) could serve in diagnose and evaluate cirrhosis in ALD. To this purpose, uEVs characterization by cryo-electron microscopy (Cryo-EM), Nanoparticle Tracking Analysis (NTA) and Western blotting (WB) was performed in a cohort of 21 controls and 21 cirrhotic patients. Then, proteomics of uEVs was carried out in a second cohort of 6 controls and 8 patients in order to identify new putative biomarkers for cirrhosis in ALD. Interestingly, uEVs concentration, size and protein composition were altered in cirrhotic patients. From a total of 1304 proteins identified in uEVs, 90 of them were found to be altered in cirrhotic patients. The results suggest that uEVs could be considered as a tool and a supplier of new biomarkers for cirrhosis in ALD, whose application would be especially relevant in chronic patients. Yet, further research is necessary to obtain more relevant result in clinical terms.

## Introduction

Alcohol is a psychoactive and toxic substance whose harmful use causes dependence, becoming one of the leading risk factors for population health worldwide. The impact of alcohol abuse is so relevant, that its prevention and treatment is included specifically by World Health Organization (WHO) as a target in the Sustainable Development Goals 3 (SDG 3), together with narcotic drugs. According to WHO, alcohol consumption contributes to 3 million deaths each year globally as well as to disabilities and poor health of millions of people. Overall, harmful use of alcohol is responsible for 5.1% of the global burden of disease 1. Indeed, alcohol abuse has an important influence on the mortality and morbidity related to more than 200 diseases and conditions 2,3. Consequently, the costs associated with alcohol amount to more than 1% of the gross national product in high and middle-income countries 1.

Alcohol is one of the most common causes of digestive diseases and, in particular, of alcoholic liver disease, or ALD 3, due to ethanol metabolism-associated products that cause hepatocellular damage 4. ALD ranges from steatosis and steatohepatitis through progressive fibrosis to cirrhosis and finally, hepatocellular cancer 5,6. More than 90% of all heavy drinkers develop fatty liver, over 10-35% of them develop severe alcoholic hepatitis and 8-20% progress in cirrhosis, while 2% of which will develop hepatocarcinoma 7. Steatosis or fatty liver disease is transient and reversible, but acute alcoholic hepatitis and liver cirrhosis are associated with high mortality (up to 50% in acute alcoholic hepatitis) 8, being the average survival time of patients with advanced liver cirrhosis between 1-2 years 9. In addition to this, over 80,000 deaths per year are attributable to alcohol-related hepatocellular carcinoma 10,11.

ALD is not usually detected until symptoms appear due to advanced disease development 12. The current diagnosis of ALD is subjected to the combination of several parameters. Firstly, screening question-based tests assess alcohol abuse and dependence 13 and biochemical parameters that include direct alcohol detection and cellular damage by alcohol intake give rise to an initial diagnostic assessment 13,14. Additionally, different predictive scores combining many biochemical parameters help obtain a more precise evaluation 14-17. Nevertheless, none of these parameters are fully efficient in diagnosis and prognosis and all of them lack of specificity and capability of truthfully discriminate ALD from other causes of liver disease, such as obesity, viral hepatitis, and exposure to other toxics 6. Hence, it is necessary to develop strategies for early and specific diagnosis of ALD according its aetiologic.

Accurate assessment of the full spectrum of ALD is challenging, particularly given the difficulty with discriminating between bland steatosis and steatohepatitis, and fibrosis severity. Liver biopsy remains the only test for precise appraisal, although it proves to be an invasive procedure that carries substantial risk and can cause complications 6,18. Nowadays, non-invasive imaging techniques such as transient elastography (TE, FibroScan®) 19, alternative sampling 20 and liquid biopsy 12 approaches are being applied in order to improve ALD diagnosis. Research on new early and specific biomarkers resulting from alcohol metabolism is also a strategy that is being followed 13,17. In ALD, much research is done by the use of blood (serum or plasma) 14. However, the use of urine offers an interesting new approach, given that it is the least invasive fluid to test, what is especially relevant in chronic or decompensated patients from whom it is often difficult to draw blood. To such extent, urinary extracellular vesicles (uEVs) could be an excellent source to look for new biomarkers 21.

EVs are round-shape vesicles that consist of a lipid bilayer containing cargo such as lipids, RNA, DNA, proteins and metabolites provided by the parental cell, indicating their origin and state 22. As EVs are secreted by all cell studied so far and can reach not only the cell environment but also different body fluids, they are being studied as sources of new biomarkers in a wide range of diseases 23,24. Liver diseases are not the exception, the involvement of EVs in physiological and pathogenic processes hold by the liver have been extensively demonstrated 25,26 and consequently, their study as biomarkers sources for diagnosis and monitoring of hepatic conditions is granted 27-29.

In this work, we have explored if uEVs associated proteins could detect alcoholic cirrhosis. For this purpose, uEVS concentration, size and EV-associated proteins have been studied, finding several alterations in patients. Remarkably, proteomics and differential analysis found alterations in patients respect to non-cirrhotic individuals in 90 proteins, which could help and complement current ALD diagnosis.

## Results

### Characterization of small urine EVs from cirrhotic patients and control individuals

In this study, uEVs obtained from 2 independent cohorts were used (Table 1, Table S1). Both of them included patients diagnosed with alcoholic cirrhosis, male and female genders and an age range comprising individuals between 18-83 or 27-73 years old, respectively.

**Figure 1:**
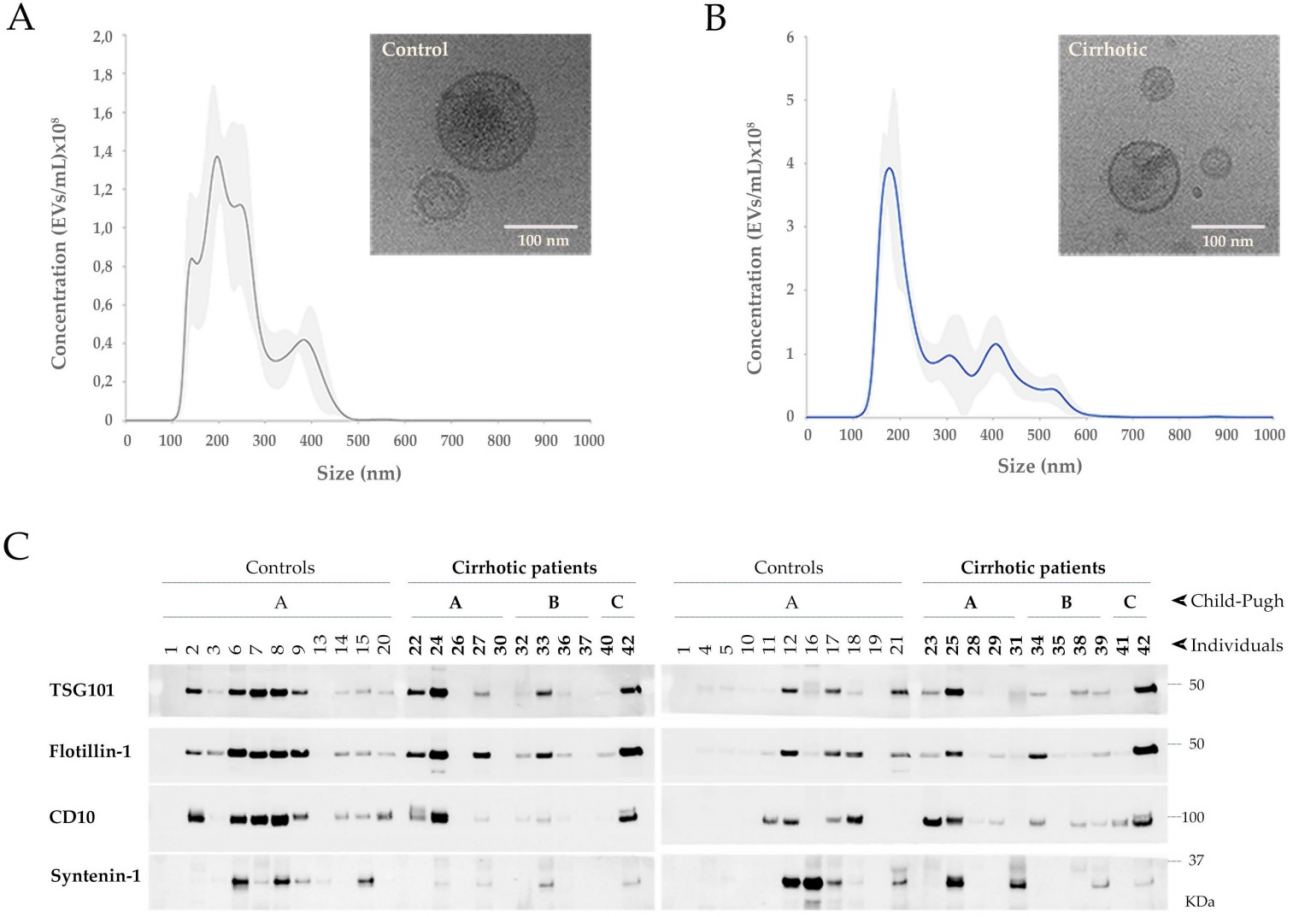
**Characterization of uEVs in the cohort 1**. A, B) Representative NTA profiles and Cryo-EM images corresponding to a representative non-cirrhotic (Control) individual and a cirrhotic patient. NTA profiles show the average values of 5 measurements and the associated standard deviation for each size in grey shadow. Cryo-EM images show illustrative uEVs. Scale bar 100 nm. C) WB for TSG101, Flotillin1, CD10 and Syntenin-1 on 15 µL of sample from 1:1000Vol_o_.

Small uEVs in the cohort 1 were characterized by cryo-electron microscopy (Cryo-EM), studied their profile population by nanoparticle tracking analysis (NTA) and biochemically analyzed by Western blotting (WB) for EV markers (Figure 1). Cryo-EM showed typical rounded vesicles in a range of 30-200 nm in size in both controls and patients. Characteristic bilayered, smooth or decorated uEVs with different electron densities were observed supporting the heterogeneity found among the EVs (Figure 1A, B). Although by cryo-EM most of the vesicles in our preparations were in range 30-200 nm, NTA characterization shows a profile corresponding to small-medium EVs (Figure 1A, B) detecting also particles bigger than 200 nm what reflects the heterogeneity of the samples. Immunoblotting analysis detected 4 out 13 proteins well-known to be associated to uEVs: TSG101, Flotillin-1, CD10 and Syntenin-1 (Figure 1C). No signal was obtained for CD63, CD81, Rab27, Caveolin-1, AQP1, AQP2, Glypican-1, Syndecan-4 and EpCAM.

**Figure 2:**
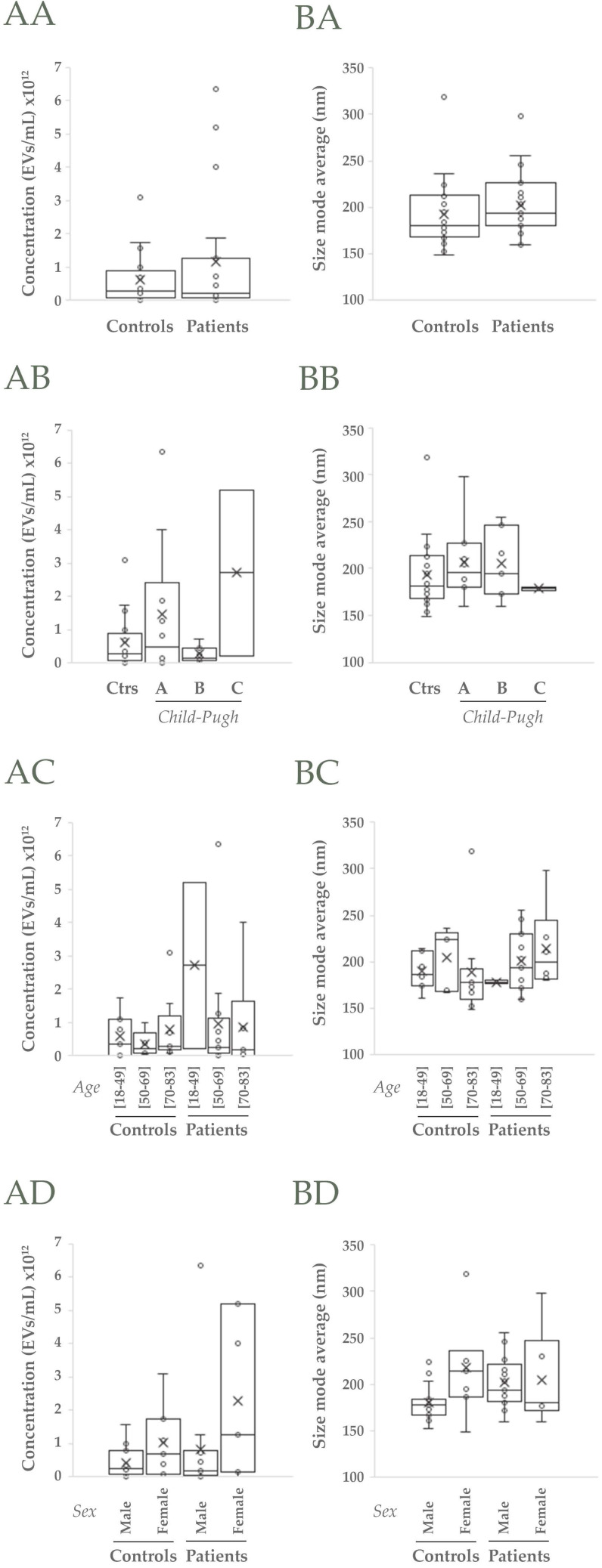
**NTA analysis of uEVs in the cohort 1.** A, B) Analysis of uEVs in terms of concentration and size (mode), respectively: AA, BA) Non-cirrhotic (Controls) *vs* cirrhotic patients (t-Student, *p*>0.05); AB, BB) Comparison among Controls, A, B and C *Child-Pugh* categories; AC, BC) Comparison among three age ranges from both Controls and patients; AD, BD) Comparison among male and female groups from both Controls and patients (ANOVA, *p*>0.05 in all cases).

Next, uEVs features were analyzed in detail to know if they could be valuable to diagnose, predict and monitor alcoholic cirrhosis. EV-concentration was observed to slightly increase in cirrhotic patients with respect to controls (*p*>0.05, Figure 2AA) due mainly to A and C *Child-Pugh* categories (*p*>0.05, Figure 2AB) and especially to the female individuals <50 years old that comprise the latest category (*p*>0.05, Figure 2AC, AD). However, in populations aged between 50-69 differences are also observed (*p*>0.05, Figure 2AC). On the other hand, the most representative EV size (size mode average) also increases in cirrhotic patients due mainly to the EV size that best represent A and B *Child-Pugh* categories and specially the male population >70 years old (*p*>0.05, Figure 2B).

**Figure 3:**
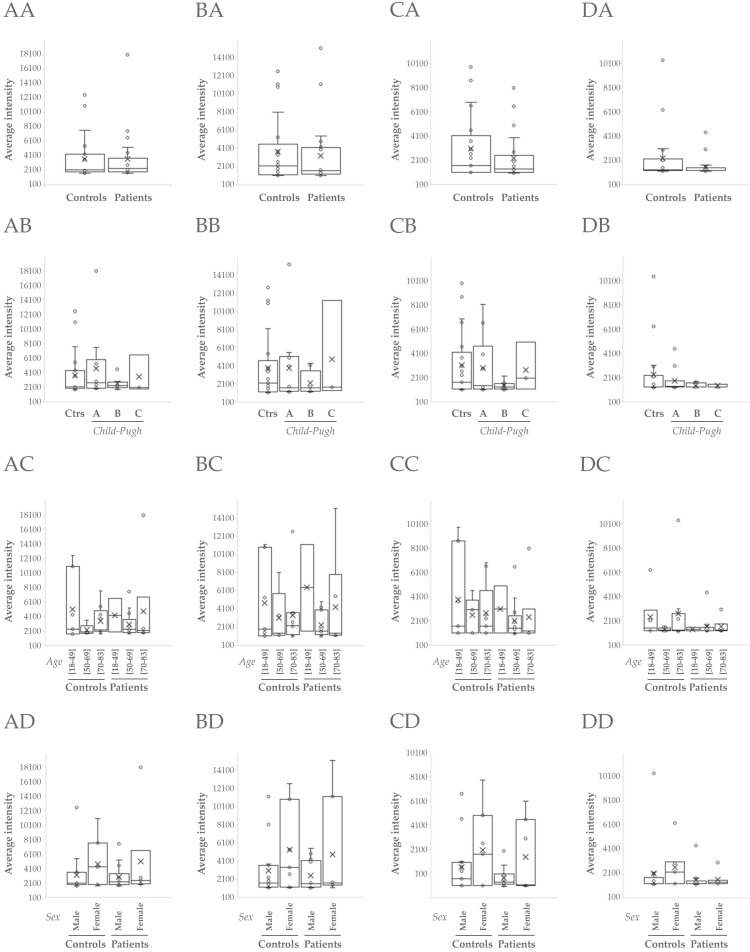
**Western Blot analysis of uEVs in the cohort 1.** A, B, C, D) Analysis of abundance of TSG101, Flotillin-1, CD10 and Syntenin-1, respectively: AA, BA, CA, DA) Non-cirrhotic (Controls) *vs* cirrhotic patients (Student's test, *p*>0.05); AB, BB, CB, DB) Comparison among Controls, A, B and C *Child-Pugh* categories; AC, BC, CC, DC) Comparison among three age ranges from both Controls and patients; AD, BD, CD, DD) Comparison among male and female groups from both Controls and patients (ANOVA, *p*>0.05 in all cases).

TSG101 and Flotillin-1 did not apparently change when compared controls *vs* patients (*p*>0.05, Figure 3AA, BA), though they increased in cirrhotic patients > 50 and >70 years old respectively (*p*>0.05, Figure 3AC, BC). CD10 and Syntenin-1 tendered to decrease in cirrhotic people, CD10 mainly in male individuals (*p*>0.05, Figure 3CD) and Syntenin-1 regardless of gender (*p*>0.05, Figure 3DD).

These results suggest that both gender and age contribute to the structure of uEVs populations and their composition. Interestingly, EV concentration and size behave differently in response to these variables.

### Proteomics analysis of small urine EVs from cirrhotic patients and control individuals

**Figure 4:**
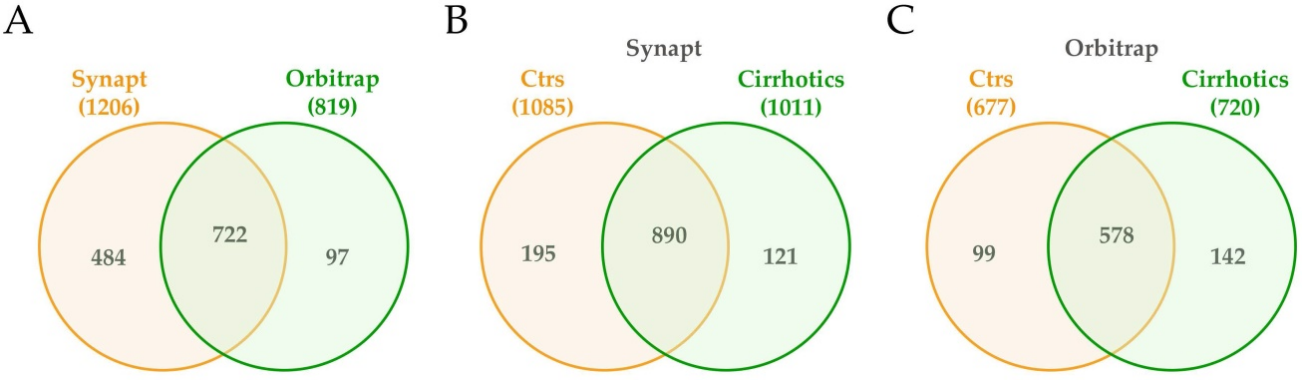
**Venn diagrams of proteins detected in uEVs from non-cirrhotic (Controls) and cirrhotic individuals by Synapt and Orbitrat acquisition strategies.** Diagrams represent the number of Uniprot/Swissprot entries identified by Mascot search engine (Matrix Science Ltd.) through Proteome Discoverer software 1.4 (Thermo).

Next, proteomic composition of small urine EVs from controls and cirrhotic patients was determined in the cohort 2 using mass spectrometry technology (Table S2). In fact, to tailor a protein list as complete as possible, both acquisition by Synapt G2Si ESI Q-Mobility-TOF (hereafter Synapt) and LTQ Orbitrap XL ETD (hereafter Orbitrap) mass spectrometers was conducted to widely cover the proteome. EVs from 6 controls and 5 cirrhotic individuals were used for Synapt data acquisition, while 3 extra cirrhotic individuals were added for Orbitrap acquisition data. Database searches identified a total of 1206 different proteins by the first method and 819 by the second, with 720 proteins in common (Figure 4A). From the 1206 Synapt proteins, 889 were common in controls and cirrhotic individuals, whereas 195 were exclusively detected in controls and 121 in patients (Figure 4B). With respect to Orbitrap method, from 819 proteins, 578 were identified in all individuals, whereas 99 solely in controls and 142 in patients (Figure 4C). The results also confirmed the presence of TSG101, Flotillin-1, CD10 and Syntenin1 EV markers detected by WB (Table S2).

### Differential analysis of the protein content of EVs from cirrhotic patients and control individuals

**Figure 5:**
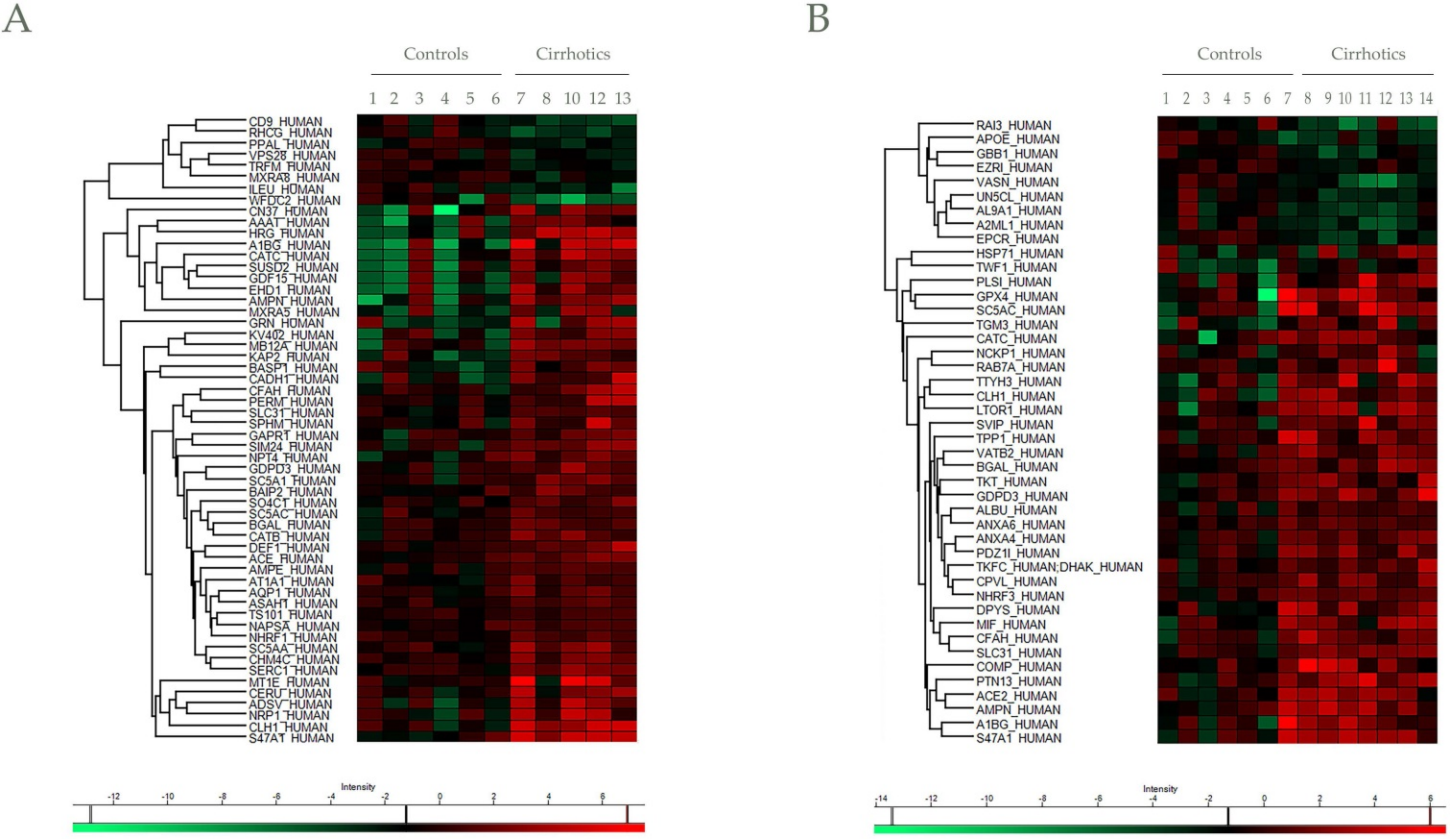
**Heat maps for differential proteins between controls and cirrhotic patients in Synapt and Orbitrap methodologies.** Hierarchical clustering of the relative abundance of A) 56 (Synapt) and B) 44 (Orbitrap) identified proteins. For a clearer representation, protein abundances were normalized against Control average values. The vertical dendrogram represents the correlation distances between protein abundance levels (Perseus program). Colors represent the relative abundance (upregulation in red, downregulation in green). Relative abundance and raw data of each protein are included in Table S2. Only proteins identified with at least two peptides at FDR<1% were considered in the analysis.

In addition to protein identification, differential protein content between EV controls and cirrhotic was also determined. Progenesis LC-MS with an ANOVA *p*-value ≤ 0.05 analysis reported a total of 59 differentially regulated proteins in cirrhotic patients *vs* controls, represented by Heatmap visualization (by Perseus) (Figure 5A,B). Among them, 10 differences were found by both mass spectrometers with different significance (Table S2), whereas 46 and 34 were unique findings of Synapt and Orbitrap systems, respectively. Importantly, a total of 20 proteins described previously as putative biomarkers for liver disease (Table 2).

**Figure 6:**
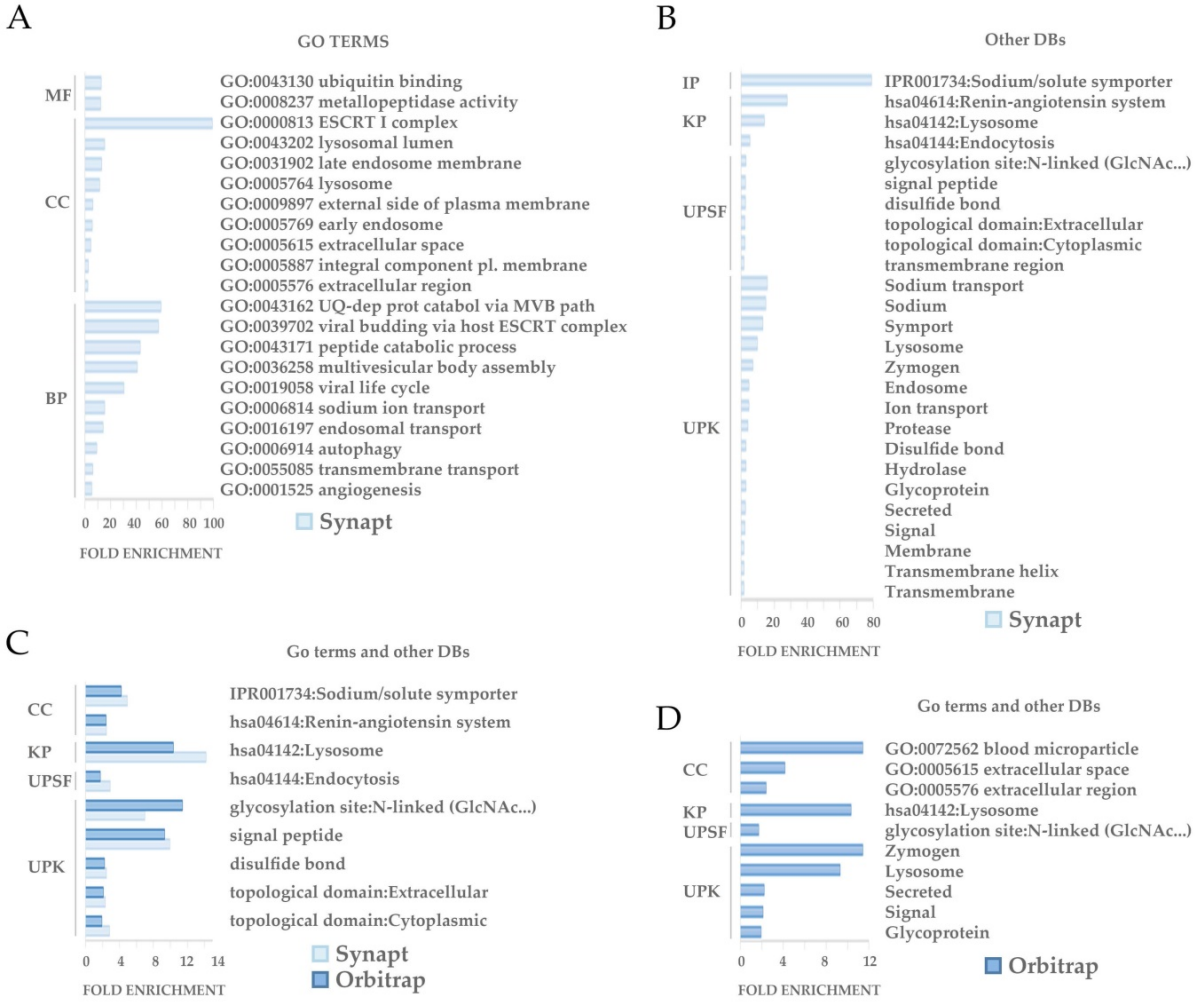
**GO enrichment analysis.** GO enrichment analysis of differentially regulated proteins between controls and patients found by Synapt and Orbitrap methodologies was performed by DAVID. The GO term or categories Molecular Function (MF), Cellular Component (CC) and Biological Process (BP) were assessed. Additionally, Interpro (IP), KEGG Pathways (KP), sequences (UPSF) and keywords (UPK) were also analyzed, considering terms with an enrichment *p*-value<0.05.

Functional analysis revealed a number of significantly enriched processes* (p*<0.05) among the set of differetially expressed proteins (Figure 6). 47 of the differentially regulated proteins detected by Synapt and 10 of the ones detected by Orbitrap were shown to be involved on these significantly enriched processes. GO analysis revealed that the differential proteins are especially involved in protein traffic processes and secretion, being the endocytic system and ESCRT machinery well represented in accordance with the EVs nature. Other functions involved glycosidation, sodium transport and angiogenesis (Figure 6A, B, C). As expected, Synapt strategy resulted in a wider list of proteins compared to Orbitrap acquisition. Accordingly, 9 out 10 enriched proceses identified after GO study on Orbitrap differential proteins were also detected by Synapt. 1 out 10, defined as blood microparticle GO cellular component and represented by ApoE, was the exception (Figure 6C).

## Discussion

Serum and plasma EVs have been largely studied as sources of biomarkers and hepatic conditions are not the exception 30,31. Indeed, they also contribute to the function in liver 26. However, in chronic patients, blood sampling becomes more problematic as the illness worsens. Thus, we have explored different features of hepatic EVs in our group, demonstrating the utility of urinary EVs (uEVs) to detect changes in the liver in response to toxicity 32,33.

In this work, we wondered if uEVs could serve as indicators of hepatic injury in alcoholic cirrhosis. We observed changes in the uEV population not only in terms of concentration as observed in previous studies with circulating EVs 27,34-37, but also in size (Figure 2). Both parameters seemed to be influenced by gender in opposite sense. In agreement with this, MELD clinical score also seems to be gender and age dependent (Table S1). That these two variables must be taken into consideration in ALD diagnosis and evaluation is a fact. WHO publishes that the percentage of men deaths attributable to alcohol and disability-adjusted lifeyears (DALYs) are more than twice compared to women 1. Indeed, older men are at the highest risk to HCC development 38.

EVs allow liquid biopsy and protect biomarkers from degradation for longer time, making them more stable in injury diagnosis and assessment. Thus, well-known EV-associated proteins could also be worthy directly studying as putative indicators. In this work, TSG101, Flotillin-1, CD10 and Syntenin-1 have been shown to behave differently, suggesting that they could represent distinct uEVs populations. TSG101 and Flotillin-1 typical EV-markers have been described that are elevated in HCC patients and associated with HCC progression 39,40, appearing TSG101 slightly upregulated in cirrhotic patients from our proteomics analysis. However, TSG101 and Flotillin-1 abundance did not apparently change in uEVs when compared controls *vs* patients (Figure 3AA, BA) by WB, though they trended to increase in cirrhotic patients > 50 and >70 years old respectively. Such difference disappears when studied men and woman separately, there being greater accumulation of TSG101 and Flotillin-1 in uEVs from women. Another classical EV-protein as CD10 or Neprilysin is known to reduce abundance and change pattern in liver biopsies with advance fibrosis or presence of lobular inflammation or extensive metastases 41. In particular, CD10 also distinguishes HCC primary tumors from secondary hepatocellular tumors 42. A decrease tendency in this protein is also observed in this study, more evident in men and clearly when compared age groups (Figure 3C). Another EV-protein considered in this study is Syntenin-1, reported to decrease in urine from patients with mitochondria disease 43. In accordance with this, Syntenin1 associated with uEV decreases with disease progression (Figure 3D).

Our results suggest that uEV proteins could act as direct biomarkers along with other more specific. Indeed, a typical EV marker, CD81, acts as receptor for hepatitis C virus and has been reported to decrease intracellularly while increasing in serum according to the severity of the disease 44,45. CD81 is associated with differentiation and metastasis of HCC 46 and, more recently, with glucose intolerance and insulin resistance, so it is suggested as an useful index to predict the risk of future metabolic disorders or the future success of efforts to control body weigh 47.

Our proteomics analysis by using Synapt and Orbitrap acquisition reported a total of 90 putative EV-associated biomarkers for ALD, including TSG101 (Table 2S). Many of them (20) have previously been reported as putative biomarkers in liver disease (Table 2). ACE and ACE2, that orchestrate together with CD10 the Renin-angiotensin system, play an important role in metabolic syndrome and liver disease development 48,49. Both partners have been extensively studied in liver disease and their serum levels are reported to correlate with the severity of disease 50-52. Lately, ACE2 has aroused great interest since it is a receptor for SARS-CoV-2 entry. The fact that it is highly expressed in the gastrointestinal tract and presents elevated levels when liver disease would explain the high susceptibility of patients to SARS-CoV-2 infection 53-55. EV- associated ACE2 has a protective activity 56,57, otherwise it can act as virus docking, endocytic pathway entry for cell infection and virus spreading 58. Indeed, ACE2 homologue, Collectrin, has been involved in intracellular trafficking and signaling 59 and AMPN, a well-known EV-component, is a coreceptor for the virus 60 and recently described in HCC promotion 61. Hence, EV-based strategies for the treatment of COVID-19 virus infection may be through inhibition of exosome biogenesis and/or EV-vaccine 62.

Renin-angiotensin system is also responsible, in part, for hypervolemic hyponatremia in cirrhosis. This syndrome is attributed to poor functional capacity of the kidneys to eliminate solute-free water, resulting in an elevated accumulation of water in relation to sodium 63. In our study, upregulation of 5 sodium-coupled transporters in secreted uEVs from cirrhotic patients was observed (SC5AC, SC5AA, NPT4, SC5A1, AT1A1), maybe contributing to sodium balance. Beside this, iron is another key element in liver disease since it is accountable for ferroptosis, an iron-dependent form of cell death characterized by the loss of lipid peroxide repair activity by glutathione peroxidase 4 (GPX4) 64. The increase in uEV-GPX4 secretion observed in our study could contribute to the whole loss of its activity when cirrhosis, becoming a putative target for therapy. Targeting ferroptosis may prevent the progression of several liver diseases, including ethanol-induced liver injury 65. Indeed, ferroptosis-inducing therapy is being applied as treatment in many cancers by using drugs such as sorafenib, erastin and RSL3 66, and new formulations with erastin-loaded exosomes are been explored 67. Other upregulated uEV-proteins involved in homeostasis of molecules are RHCG, that plays a major role in transporting ammonia and accumulates in acute liver failure, resulting in brain damage 68,69; and GDPD3, responsible for lysophosphatidic acid synthesis that, in turns, is involved in liver disease development 70-72.

Some uEVs proteins found in the proteomics analysis to be differentially regulated in cirrhotic patients such CADH1, CATC, TKT and VASH are known to promote HCC 73-76. Others, such as WFDC2, GRN, SVIP and BGAL are reported to exert control on the disease 77-79. In particular, BGAL is a marker of senescence that takes place during the progression of cirrhosis and HCC as part of the antitumoral response 80,81.

Multiple lines of evidence indicate that many biomarkers could change inversely proportional to the disease progression. COMP, for example, was found in our proteomics analysis to be upregulated. Importantly, it has been reported that the amount of this protein increases in early cirrhosis and then, decreases in advanced stages ^82^. Finding biomarkers that assess the course of liver disease or stages and indicate the underlying etiology is highly challenging. In this line, impact of alcohol on EV contents in alcohol-related or xenobiotic exposure is an under-studied area 29,83,84. Interestingly, we found MIF, that play an important role in ALD development, to be upregulated in uEV 85-87. Indeed, EV-MIF was described to be necessary for pre-metastatic niche development in liver 88.

In this pilot study, we have explored uEV in alcoholic cirrhosis, finding evidence that supports their consideration in diagnosis and assessment of the disease. However, given the high inter-individual variability found in this work and EV heterogeneity, a higher number of samples would be necessary to truly establish uEV as a source of biomarkers for cirrhosis detection and diagnosis.

## Materials and Methods

### Experimental design

The summary of the experimental workflow can be found in the Graphical Abstract.

### Patient samples

Urine samples and data from patients included in this study were provided by the Basque Biobank (www.biobancovasco.org), BIOEF, Basurto University Hospital) upon informed consent and with evaluation and appropriate approval of the Ethical and Scientific Committees (code CEIC 10-01). Both controls and patients were evaluated for ALD. Patients included in the study were diagnosed with hepatic cirrhosis by means of clinical, analytic and echography findings, with an alcohol consumption of 60 g/d in men and 40 g/d in women (Table S1). Exclusion criteria comprise other causes of liver diseases, including VHB or VHC infection with alcohol consumption.

Controls did not take any drug during the study. Cirrhotic patients took medication for prevention or treatment of cirrhosis-associated decompensations (ascites, variceal bleeding and hepatic encephalopathy). These include diuretics, such as spironolactone y furosemide; laxative-lactulose o beta-blockers as propranolol. In all cases, the first urine of the morning was collected for uEVs isolation. Controls made normal life with a water consumption from 1.5 to 3 L per day, while cirrhotic patients are recommended to drink 1.5 L per day.

Between 6-150 mL of urine was collected by spontaneous micturition, centrifuged at 1,500 × g 5 min, filtered through a 0.22 μm-pore membrane and immediately frozen at -80 ºC until processing for uEVs purification.

In this study, two independent cohorts were used (Table 1, Table S1). Both of them included patients diagnosed with different stages of alcoholic cirrhosis, male and female genders and an age range between 18-83 years or 27-73 individuals respectively. The cohort 1 consisted of 21 controls and 21 cirrhotic patients where uEVs were characterized and analysed by Cryo Electron microspopy (Cryo-EM), Nanoparticle Tracking Analysis (NTA) and EV-markers abundance by western blotting (WB). The cohort 2 comprised urine samples donated by 6 controls and 8 patients where uEVs were submitted for proteomics analysis. For this purpose, two proteomics approaches were used by means of Synapt G2Si ESI Q-Mobility-TOF (Waters) and LTQ Orbitrap XL ETD (Thermo) spectrometers.

### Urine extracellular vesicle isolation and use

To isolate EVs from urine, the stored samples at -80 ºC were thawed overnight at 4 ºC, centrifuged at 10,000 × g for 30 min and the supernatant ultra-centrifuged at 100,000 × g for 75 min. Final pellet (P100) was re-suspended in PBS. In all cases, a volume of 1:1000 of PBS respect to the starting urine volume was employed, aliquots generated if necessary and kept at -80 °C for further analysis.

For the first cohort, 20 µL were loaded for Western blotting analysis, 5 µ for NTA and 5 µL for Cryo-EM. In the case of the second cohort, 9/10 of EVs sample volume was utilized for proteomics acquisition.

### Cryo-Electron Microscopy

For negative staining, vesicles were adsorbed onto glow-discharged Formvar-Carbon Niquel grids, washed with distilled water and stained with freshly prepared 2% uranyl acetate in aqueous suspension. Negative stained samples were imaged at room temperature using a JEM-1230 transmission electron microscope (JEOL) equipped with a thermionic tungsten filament and operated at an acceleration voltage of 120 kV. Images were taken using the ORIUS SC1000 (4008 × 2672 pixels) cooled slow-scan CCD camera (GATAN). For cryo-electron microscopy, EV preparations were directly adsorbed onto glow-discharged holey carbon grids (QUANTIFOIL, Germany). Grids were blotted at 95% humidity and rapidly plunged into liquid ethane with the aid of a VITROBOT (Maastricht Instruments BV, The Netherlands). Vitrified samples were imaged at liquid nitrogen temperature using a JEM-2200FS/CR transmission cryo-electron microscope (JEOL, Japan) equipped with a field emission gun and operated at an acceleration voltage of 200 kV.

### Nanoparticle tracking analysis (NTA)

Size distribution of the uEVs preparations was determined by measuring the Brownian motion using a NanoSight LM10 system equipped with a fast video capture and particle-tracking software (Malvern, UK). Pre- and post-acquisition settings were maintained the same for all the samples and each video was analyzed to give the mode for particle size as well as an estimate of the particle concentration. Each sample was acquired 5 times. Then, an average curve was calculated for each sample. Comparative analysis between or among groups was performed by means of Student's test or ANOVA respectively.

### Western blot (WB) analysis

For each sample, 15 µL of PBS-resuspended EVs were mixed with NuPAGE LDS Sample Buffer (Invitrogen by Thermo Scientific) for direct lysis. The samples were incubated for 5 min at 37 °C, 10 min at 65 °C, and 15 min at 95 °C, centrifuged at 20 000 g for 15 min and supernatant separated on NuPAGE 4-12% pre-casted gels (Invitrogen by Thermo Scientific). Proteins were transferred to a PVDF membrane (Millipore by Merck) that was then blocked for 1 h in 5% milk and 0.05% Tween-20 in PBS. Then, the membrane was incubated overnight at 4 °C with the primary antibody, followed by PBS washing before application of the corresponding secondary HRP-conjugated antibody. Chemiluminescent bands were detected with Pierce™ ECL Plus Western Blotting Substrate (Pierce by Thermo Scientific).

Mouse monoclonal antibodies were purchased from the following vendors: mouse monoclonal antibody against TSG101 (clone 4A10) was obtained from Abcam, against Flotillin-1 (clon 18) and Rab27 (clon 20) from BD Biosciences, for CD10 (Neprilysin, clon F-4) and Glypican-1 (A-10) from Santa Cruz Biotechnology, Inc., and for CD63 (clon H5C6), CD81 (clon JS81) and EpCAM (clon G8.8) from Hybridoma Bank. Rabbit polyclonal antibody against Syntenin-1 (clon C2C3) was purchased from GeneTex, Caveolin-1 (ab2910) and Syndecan4 (ab24511) from Abcam, AQP1 (A5560) and AQP2 (A7310) from Sigma.

Signal for each protein was quantified by densitometry by using the ImageQuant image software. Comparative analysis between or among groups was performed by means of Student's t-test or ANOVA respectively.

### Proteomics

#### In solution digestion

Protein was extracted using 7M urea, 2M thiourea, 4% CHAPS. Samples were incubated for 30 min at RT under agitation and digested following the filter-aided FASP protocol described by Wisniewski et al 89 with minor modifications. Trypsin was added to a trypsin:protein ratio of 1:10, and the mixture was incubated overnight at 37 ºC, dried out in a RVC2 25 speedvac concentrator (Christ), and resuspended in 0.1% FA.

#### LC-MS analysis

LC was performed using a NanoAcquity nano-HPLC (Waters), equipped with a Waters BEH C18 nano-column (200 mm × 75 um ID, 1.8 um). A chromatographic ramp of 120 min (5 to 60% ACN) was used with a flow rate of 300 nl/min. Mobile phase A was water containing 0.1% v/v formic acid, while mobile phase B was ACN containing 0.1% v/v formic acid.

Samples were acquired using two different mass spectrometers. On the one hand, a Synapt G2Si ESI Q-Mobility-TOF spectrometer (Waters) equipped with an ion mobility chamber (T-Wave-IMS) for high-definition data acquisition analyses was used. All analyses were performed in positive mode ESI. Data were post-acquisition lock mass corrected using the double charged monoisotopic ion of [Glu1]-Fibrinopeptide B. Accurate mass LC-MS data were collected in HDDA mode that enhances signal intensities using the ion mobility separation step. On the other hand, sample was also loaded onto an LTQ Orbitrap XL ETD (Thermo). This mass spectrometer automatically switched between MS and MS/MS acquisition in DDA mode. Full MS scan survey spectra (m/z 400-2000) were acquired in the orbitrap with mass resolution of 30000 at m/z 400. After each survey scan, the six most intense ions above 1000 counts were sequentially subjected to collision-induced dissociation (CID) in the linear ion trap. Precursors with charge states of 2 and 3 were specifically selected for CID. Peptides were excluded from further analysis during 60 s using the dynamic exclusion feature.

### Bioinformatics

#### Database searches and protein identification

Searches were carried out using Mascot search engine (Matrix Science Ltd.) through Proteome Discoverer software 1.4 (Thermo). Orbitrap RAW files were directly loaded into the program, whereas mgf files generated by DataAnalysis software (Bruker) were used for timsTOF searches. Orbitrap searches were carried out with precursor and fragment tolerances of 10 ppm and 0.5 Da, whereas 50 ppm and 0.05 Da were used for TIMS TOF runs. A database consisting of human entries (Uniprot/Swissprot) was used for the searches. Only proteins identified with at least two peptides at FDR<1% in at least two sample replicas and not present in the negative control were considered for further analysis. InteractiveVenn (http://www.interactivenn.net/) was used for making Venn diagrams.

#### Differential protein content analysis

Progenesis LC-MS (version 4.2.7207.22925, Nonlinear Dynamics) was used for the label-free differential protein content analysis. One of the runs was used as the reference to which the precursor masses in all other samples were aligned to. Only features comprising charges of 2+ and 3+ were selected. The raw abundances of each feature were automatically normalized and logarithmized against the reference run. Samples were grouped in accordance with the comparison being performed, and an ANOVA analysis was performed. A peak list containing the information of all the features was generated and exported to the Mascot search engine (Matrix Science Ltd.). This file was searched against a Uniprot/Swissprot database, and the list of identified peptides was imported back to Progenesis LC-MS. Protein quantitation was performed based on the three most intense non-conflicting peptides (peptides occurring in only one protein), except for proteins with only two non-conflicting peptides. The significance of expression changes was tested at protein level, and proteins with an ANOVA *p*-value ≤ 0.05 were selected for further analyses. Heatmaps were generated using Perseus software 90.

#### Functional analysis

GO enrichment analysis was carried out using the DAVID online tool (http://david.abcc.ncifcrf.gov/summary.jsp) 91. DAVID is a GO Term annotation and enrichment analysis tool used to highlight the most relevant GO terms associated with a given gene list. A Fisher Exact test is used in order to determine whether the proportion of genes considered into certain GO term or categories differ significantly between the dataset and the background. Biological Process (BP), Molecular Function (MF) and Cellular Component (CC) categories were assessed. Additionally, KEGG Pathways, keywords, sequences, and Interpro and Smart databases were also analyzed, considering terms with an enrichment *p*-value<0.05.

## Conclusions

Accurate diagnosis and assessment of ALD is challenging, particularly because of the difficulty of identifying early stages of the disease before fibrosis progresses to develop cirrhosis and/or HCC. Additionally, there is no unique clinical presentation of ALD that can be distinguished with certainty from other forms of liver disease. Proteomics, genomics and metabolomics approaches could also help identify the etiology of liver disease 92,93 and, in case of ALD, even understand the detoxification mechanisms of alcohol 94. In this line, liquid biopsy approaches, as EVs, provide great knowledge and could become useful tools. In our study, changes in concentration and size, as well as in composition have been observed in uEVs from cirrhotic patients. Supporting also the value of EVs in ALD diagnosis, Sehrawat et al., 2020 made the observation that circulating EV concentration and sphingolipid cargo signature could diagnose and differentiate alcoholic hepatitis (AH) in heavy drinkers from decompensated alcoholic cirrhosis (AC), and other etiologies of end-stage liver disease (ESLD). Other authors also described changes when ALD in circulating EVs concentration 27,35-37, certain miRNAs 27,37,95 and hepatic proteins such as ALB, HP, FGB, CYP2E1, 2A, 1A1/2, 4B 35,36. For HCC diagnosis, new EVs-based approaches that allow easy and standardized use in clinical settings are being studied 96. The proteins detected to be differently regulated in cirrhotic patients in our study are not specifically expressed in liver. Indeed, some deregulated proteins could result as a consequence of the disease in other tissues as these patients could have co-morbilities. May be a more extensive study hopefully allows the scenario to identify a specific marker for hepatic EVs.

Sampling selection and management in liquid biopsy is key to diagnosis. In this sense, urine could become an appropriate alternative for sampling in diagnosis and EV-isolation in chronic patients, although this fluid presents several limitations that must be solved. We have found several obstacles along this work, such as the impossibility of quantifying the amount of protein in EV-isolates because of the urine pH, leading to explain the data on the basis of sample volume instead of on protein concentration. Furthermore, the lack of standardized protocols for sample collection introduces some uncontrollable variables. Apart from addressing these questions, also more numerous cohorts of individuals, containing similar number of individuals of either gender, in the distinct ranges of age and Child-Pugh categories for cirrhosis are needed to obtain more definitive conclusions.

## Supplementary Material

Supplementary table S1.

Supplementary table S2.

## Figures and Tables

**Table 1:** Clinical samples. Cohorts 1 and 2 description according to cirrhosis stages, gender and age

	No. individuals	Male individuals	Female individuals	HCC^1^ (males)	MELD score	Age (years)^2^	Age 18-49^3^	Age 50-69^3^	Age 70-83^3^
**Cohort 1**									
Non-cirrhotic (Controls)	21	14	7	0	6-12	18-81	7	5	9
Patients A^4^	10	6	4	1	8-18	52-83	0	4	6
Patients B^4^	8	7	1	1	14-27	50-69	0	8	0
Patients C^4^	3	1	2	0	11-24	37-58	2	1	0
**Cohort 2**									
Non-cirrhotic (Controls)	6	1	5	0	6-7	27-43	6	0	0
Patients A^4^	4	3	1	0	6-10	58-73	0	3	1
Patients B^4^	3	2	1	1	8-24	43-48	2	1	0
Patients C^4^	1	0	1	0	19	46	1	0	0

^1^ HCC cases were only in male individuals. ^2^ Ranges of age (years). ^3^ Number of individuals in the corresponding range of age. Cirrhosis stages according to ^4^ *Child-Pugh* categories (A, B or C).

**Table 2:** Proteins found differentially regulated in uEVs from cirrhotic patients that were previously described as putative biomarkers for liver disease

Accession	Specie	Molecule	Disease	Sample	References
CLH1_HUMAN^1^	Human	Protein	Cirrhosis	Liver biopsy	97
A1BG_HUMAN^1^	Human	Protein	Chronic alcoholics liver damage	Blood lymphocytes	98
	Rat	Protein	Regeneration model	Liver biopsy	99
	Mice	Protein	Adenoma model	Serum	100
AMPN_HUMAN^1^	Human	Protein	HCC	Liver biopsy	101
GDPD3_HUMAN^1^	Human	mRNA	Liver disease	Liver biopsy	72
ACE_HUMAN^2^	Human	Protein	ALD	Serum	102
HRG_HUMAN^2^	Human	Protein	Cirrhosis	Liver biopsy	103
DEF1_HUMAN^2^	Human	Protein	Acute-non chronic liver failure	Serum	104
CATB_HUMAN^2^	Human	Protein	Cirrhosis, HCC	Serum	105
AMPE_HUMAN^2^	Human	Activity	Cirrhosis	Serum	106
MT1E_HUMAN^2^	Human	mRNA	ALD	Blood	107
GDF15_HUMAN^2^	Human	Protein	Chronic liver disease	Serum	108
	Mice	Protein/mRNA	ALD model	Serum/Liver biopsy	109
NRP1_HUMAN^2^	Human	Protein	HCC	Liver biopsy	110
SUSD2_HUMAN^2^	Human	Protein/mRNA	HCC	Liver biopsy	111
CERU_HUMAN^2^	Human	Protein	Wilson disease	Serum	112
ACE2_HUMAN^3^	Human	Protein	Liver disease	Serum	50-52
ALBU_HUMAN^3^	Human	Protein	Liver disease	Serum	113,114
MIF_HUMAN^3^	Human	Protein	ALD	Serum/Liver biopsy	86,115
ANXA6_HUMAN^3^	Human	Protein/mRNA	HCC	Liver biopsy	116
EPCR_HUMAN^3^	Human	Protein	Chronic liver disease	Plasma	117
RAI3_HUMAN^3^	Human	Protein/mRNA	HCC	Serum	118
VASN_HUMAN^3^	Human	Protein	HCC	Serum	119
COMP_HUMAN^3^	Human	Protein	Cirrhosis, risk of HCC	Serum	120

^1^ Proteins detected to be deregulated by Synapt and Orbitrap, ^2^only by Synapt or only by ^3^Orbitrap acquisition.

## Abbreviations

EVs: extracellular vesicles
uEVs: urinary EVs
ALD: alcoholic liver disease
HCC: hepatocarcinoma
AH: alcoholic hepatitis
AC: alcoholic cirrhosis
ESLD: end-stage liver disease
DALYs: disability-adjusted lifeyears
WHO: World Health Organization
SDG 3: Sustainable Development Goals
TE: transient elastography (FibroScan®)
Cryo-EM: cryo-electron microscopy
NTA: Nanoparticle Tracking Analysis
WB: Western blotting

## References

[1] WHO. Global status report on alcohol and health 2018 (World Health Organization, 2018

[2] Shield KD, Parry C, Rehm J (2013). Chronic diseases and conditions related to alcohol use. Alcohol Res.

[3] Rehm J, Gmel GE Sr, Gmel G (2017). The relationship between different dimensions of alcohol use and the burden of disease-an update. Addiction.

[4] Gao B, Bataller R (2011). Alcoholic liver disease: pathogenesis and new therapeutic targets. Gastroenterology.

[5] Mathurin P, Bataller R (2015). Trends in the management and burden of alcoholic liver disease. J Hepatol.

[6] Thursz M, Gual A, Lackner C (2018). EASL Clinical Practice Guidelines: Management of alcohol-related liver disease. J Hepatol.

[7] Seitz HK, Mueller S Alcoholic Liver Disease. In: Springer B, Heidelberg. Clinical Hepatology2010.

[8] Tsochatzis EA, Bosch J, Burroughs AK (2014). Liver cirrhosis. Lancet.

[9] Bruha R, Dvorak K, Petrtyl J (2012). Alcoholic liver disease. World J Hepatol.

[10] Rehm J, Mathers C, Popova S, Thavorncharoensap M, Teerawattananon Y, Patra J (2009). Global burden of disease and injury and economic cost attributable to alcohol use and alcohol-use disorders. Lancet.

[11] Dugum M, McCullough A (2015). Diagnosis and Management of Alcoholic Liver Disease. J Clin Transl Hepatol.

[12] Torruellas C, French SW, Medici V (2014). Diagnosis of alcoholic liver disease. World J Gastroenterol.

[13] Addolorato G, Vassallo GA, Mirijello A, Gasbarrini A (2020). Diagnosis and Management of Alcohol Use Disorder in Patients with Liver Disease: Lights and Shadows. Neurotherapeutics.

[14] Seitz HK, Bataller R, Cortez-Pinto H (2018). Alcoholic liver disease. Nat Rev Dis Primers.

[15] Rudler M, Mouri S, Charlotte F (2015). Validation of AshTest as a Non-Invasive Alternative to Transjugular Liver Biopsy in Patients with Suspected Severe Acute Alcoholic Hepatitis. PLoS One.

[16] Hadefi A, Degré D, Trépo E, Moreno C (2020). Noninvasive diagnosis in alcohol-related liver disease. Health Sci Rep. Vol 3: © 2020 The Authors. Health Science Reports published by Wiley Periodicals, Inc.

[17] Gala KS, Vatsalya V (2020). Emerging Noninvasive Biomarkers, and Medical Management Strategies for Alcoholic Hepatitis: Present Understanding and Scope. Cells.

[18] Bianchi L (2001). Liver biopsy in elevated liver functions tests? An old question revisited. J Hepatol.

[19] Lupsor-Platon M, Badea R (2015). Noninvasive assessment of alcoholic liver disease using unidimensional transient elastography (Fibroscan(®)). World J Gastroenterol.

[20] Kummer N, Lambert WE, Samyn N, Stove CP (2016). Alternative sampling strategies for the assessment of alcohol intake of living persons. Clin Biochem.

[21] Svenningsen P, Sabaratnam R, Jensen BL (2020). Urinary extracellular vesicles: Origin, role as intercellular messengers and biomarkers; efficient sorting and potential treatment options. Acta Physiol (Oxf).

[22] Yáñez-Mó M, Siljander PR, Andreu Z (2015). Biological properties of extracellular vesicles and their physiological functions. J Extracell Vesicles.

[23] González E, Falcón-Pérez JM (2015). Cell-derived extracellular vesicles as a platform to identify low-invasive disease biomarkers. Expert Rev Mol Diagn.

[24] Simeone P, Bologna G, Lanuti P (2020). Extracellular Vesicles as Signaling Mediators and Disease Biomarkers across Biological Barriers. Int J Mol Sci.

[25] Szabo G, Momen-Heravi F (2017). Extracellular vesicles in liver disease and potential as biomarkers and therapeutic targets. Nat Rev Gastroenterol Hepatol.

[26] Azparren-Angulo M, Royo F, Gonzalez E Extracellular vesicles in hepatology: Physiological role, involvement in pathogenesis, and therapeutic opportunities. Pharmacol Ther. 2020:107683.

[27] Momen-Heravi F, Saha B, Kodys K, Catalano D, Satishchandran A, Szabo G (2015). Increased number of circulating exosomes and their microRNA cargos are potential novel biomarkers in alcoholic hepatitis. J Transl Med.

[28] Hernández A, Arab JP, Reyes D (2020). Extracellular Vesicles in NAFLD/ALD: From Pathobiology to Therapy. Cells.

[29] Cho YE, Song BJ, Akbar M, Baek MC (2018). Extracellular vesicles as potential biomarkers for alcohol- and drug-induced liver injury and their therapeutic applications. Pharmacol Ther.

[30] Povero D, Yamashita H, Ren W (2020). Characterization and Proteome of Circulating Extracellular Vesicles as Potential Biomarkers for NASH. Hepatol Commun.

[31] Uzzaman A, Zhang X, Qiao Z (2020). Discovery of small extracellular vesicle proteins from human serum for liver cirrhosis and liver cancer. Biochimie.

[32] Conde-Vancells J, Rodriguez-Suarez E, Gonzalez E (2010). Candidate biomarkers in exosome-like vesicles purified from rat and mouse urine samples. Proteomics Clin Appl.

[33] Rodriguez-Suarez E, Gonzalez E, Hughes C (2014). Quantitative proteomic analysis of hepatocyte-secreted extracellular vesicles reveals candidate markers for liver toxicity. Journal of Proteomics.

[34] Payancé A, Silva-Junior G, Bissonnette J (2018). Hepatocyte microvesicle levels improve prediction of mortality in patients with cirrhosis. Hepatology.

[35] Cho YE, Mezey E, Hardwick JP, Salem N Jr, Clemens DL, Song BJ (2017). Increased ethanol-inducible cytochrome P450-2E1 and cytochrome P450 isoforms in exosomes of alcohol-exposed rodents and patients with alcoholism through oxidative and endoplasmic reticulum stress. Hepatol Commun.

[36] Cho YE, Im EJ, Moon PG, Mezey E, Song BJ, Baek MC (2017). Increased liver-specific proteins in circulating extracellular vesicles as potential biomarkers for drug- and alcohol-induced liver injury. PLoS One.

[37] Eguchi A, Lazaro RG, Wang J (2017). Extracellular vesicles released by hepatocytes from gastric infusion model of alcoholic liver disease contain a MicroRNA barcode that can be detected in blood. Hepatology.

[38] Yi SW, Choi JS, Yi JJ, Lee YH, Han KJ (2018). Risk factors for hepatocellular carcinoma by age, sex, and liver disorder status: A prospective cohort study in Korea. Cancer.

[39] Liu Z, Tian Z, Cao K (2019). TSG101 promotes the proliferation, migration and invasion of hepatocellular carcinoma cells by regulating the PEG10. J Cell Mol Med.

[40] Zhang SH, Wang CJ, Shi L (2013). High Expression of FLOT1 Is Associated with Progression and Poor Prognosis in Hepatocellular Carcinoma. PLoS One.

[41] Shousha S, Gadir F, Peston D, Bansi D, Thillainaygam AV, Murray-Lyon IM (2004). CD10 immunostaining of bile canaliculi in liver biopsies: change of staining pattern with the development of cirrhosis. Histopathology.

[42] Singha J, Khan K, Chatterjee S (2018). Diagnostic utility of CD10 immunohistochemical staining on cellblock in differentiating hepatocellular carcinoma from secondary malignancies of liver. Indian J Pathol Microbiol.

[43] Hall AM, Vilasi A, Garcia-Perez I (2015). The urinary proteome and metabonome differ from normal in adults with mitochondrial disease. Kidney Int.

[44] Welker MW, Reichert D, Susser S (2012). Soluble serum CD81 is elevated in patients with chronic hepatitis C and correlates with alanine aminotransferase serum activity. PLoS One.

[45] Inoue G, Horiike N, Onji M (2001). The CD81 expression in liver in hepatocellular carcinoma. Int J Mol Med.

[46] Mazzocca A, Liotta F, Carloni V (2008). Tetraspanin CD81-regulated cell motility plays a critical role in intrahepatic metastasis of hepatocellular carcinoma. Gastroenterology.

[47] Oguri Y, Shinoda K, Kim H (2020). CD81 Controls Beige Fat Progenitor Cell Growth and Energy Balance via FAK Signaling. Cell.

[48] Simões ESAC, Miranda AS, Rocha NP, Teixeira AL (2017). Renin angiotensin system in liver diseases: Friend or foe?. World J Gastroenterol.

[49] Sansoè G, Aragno M, Wong F (2020). Pathways of hepatic and renal damage through non-classical activation of the renin-angiotensin system in chronic liver disease. Liver Int.

[50] Miranda AS, Simões ESAC (2017). Serum levels of angiotensin converting enzyme as a biomarker of liver fibrosis. World J Gastroenterol.

[51] Casey S, Schierwagen R, Mak KY (2019). Activation of the Alternate Renin-Angiotensin System Correlates with the Clinical Status in Human Cirrhosis and Corrects Post Liver Transplantation. J Clin Med.

[52] Ye G, Qin Y, Lu X (2015). The association of renin-angiotensin system genes with the progression of hepatocellular carcinoma. Biochem Biophys Res Commun.

[53] Boettler T, Marjot T, Newsome PN (2020). Impact of COVID-19 on the care of patients with liver disease: EASL-ESCMID position paper after 6 months of the pandemic. JHEP Rep.

[54] Konturek PC, Harsch IA, Neurath MF, Zopf Y (2020). COVID-19 - more than respiratory disease: a gastroenterologist's perspective. J Physiol Pharmacol.

[55] Fondevila MF, Mercado-Gómez M, Rodríguez A (2020). Obese patients with NASH have increased hepatic expression of SARS-CoV-2 critical entry points. J Hepatol.

[56] Zhang C, Wang J, Ma X (2018). ACE2-EPC-EXs protect ageing ECs against hypoxia/reoxygenation-induced injury through the miR-18a/Nox2/ROS pathway. J Cell Mol Med.

[57] Wang J, Chen S, Bihl J (2020). Exosome-Mediated Transfer of ACE2 (Angiotensin-Converting Enzyme 2) from Endothelial Progenitor Cells Promotes Survival and Function of Endothelial Cell. Oxid Med Cell Longev.

[58] Hassanpour M, Rezaie J, Nouri M, Panahi Y (2020). The role of extracellular vesicles in COVID-19 virus infection. Infect Genet Evol.

[59] Lambert DW, Clarke NE, Turner AJ (2010). Not just angiotensinases: new roles for the angiotensin-converting enzymes. Cell Mol Life Sci.

[60] Park J, Jeong D, Chung YW, Kim DH, Cheon JH, Ryu JH (2020). Quantitative Proteomic Analysis of the Expression of SARS-CoV-2 Receptors in the Gut of Patients with Chronic Enterocolitis. Yonsei Med J.

[61] Zhao Y, Wu H, Xing X (2020). CD13 Induces Autophagy to Promote Hepatocellular Carcinoma Cell Chemoresistance Through the P38/Hsp27/CREB/ATG7 Pathway. J Pharmacol Exp Ther.

[62] Inal JM (2020). Decoy ACE2-expressing extracellular vesicles that competitively bind SARS-CoV-2 as a possible COVID-19 therapy. Clin Sci (Lond).

[63] Alukal JJ, John S, Thuluvath PJ (2020). Hyponatremia in Cirrhosis: An Update. Am J Gastroenterol.

[64] Dixon SJ, Lemberg KM, Lamprecht MR (2012). Ferroptosis: an iron-dependent form of nonapoptotic cell death. Cell.

[65] Mao L, Zhao T, Song Y (2020). The emerging role of ferroptosis in non-cancer liver diseases: hype or increasing hope?. Cell Death Dis.

[66] Capelletti MM, Manceau H, Puy H, Peoc'h K (2020). Ferroptosis in Liver Diseases: An Overview. Int J Mol Sci.

[67] Bebber CM, Müller F, Prieto Clemente L, Weber J, von Karstedt S (2020). Ferroptosis in Cancer Cell Biology. Cancers (Basel).

[68] Wang W, Lu H, Lu X (2018). Effect of tumor necrosis factor-α on the expression of the ammonia transporter Rhcg in the brain in mice with acute liver failure. J Neuroinflammation.

[69] Aldridge DR, Tranah EJ, Shawcross DL (2015). Pathogenesis of hepatic encephalopathy: role of ammonia and systemic inflammation. J Clin Exp Hepatol.

[70] Kaffe E, Magkrioti C, Aidinis V (2019). Deregulated Lysophosphatidic Acid Metabolism and Signaling in Liver Cancer. Cancers (Basel).

[71] Yoo HJ, Jung KJ, Kim M (2019). Liver Cirrhosis Patients Who Had Normal Liver Function Before Liver Cirrhosis Development Have the Altered Metabolic Profiles Before the Disease Occurrence Compared to Healthy Controls. Front Physiol.

[72] Key CC, Bishop AC, Wang X (2020). Human GDPD3 overexpression promotes liver steatosis by increasing lysophosphatidic acid production and fatty acid uptake. J Lipid Res.

[73] Nakagawa H, Hikiba Y, Hirata Y (2014). Loss of liver E-cadherin induces sclerosing cholangitis and promotes carcinogenesis. Proc Natl Acad Sci U S A.

[74] Mirea AM, Tack CJ, Chavakis T, Joosten LAB, Toonen EJM (2018). IL-1 Family Cytokine Pathways Underlying NAFLD: Towards New Treatment Strategies. Trends Mol Med.

[75] Qin Z, Xiang C, Zhong F (2019). Transketolase (TKT) activity and nuclear localization promote hepatocellular carcinoma in a metabolic and a non-metabolic manner. J Exp Clin Cancer Res.

[76] Huang A, Dong J, Li S (2015). Exosomal transfer of vasorin expressed in hepatocellular carcinoma cells promotes migration of human umbilical vein endothelial cells. Int J Biol Sci.

[77] Bruno S, Pasquino C, Herrera Sanchez MB (2020). HLSC-Derived Extracellular Vesicles Attenuate Liver Fibrosis and Inflammation in a Murine Model of Non-alcoholic Steatohepatitis. Mol Ther.

[78] Yoo W, Lee J, Noh KH (2019). Progranulin attenuates liver fibrosis by downregulating the inflammatory response. Cell Death Dis.

[79] Jia D, Wang YY, Wang P (2019). SVIP alleviates CCl(4)-induced liver fibrosis via activating autophagy and protecting hepatocytes. Cell Death Dis.

[80] Wiemann SU, Satyanarayana A, Tsahuridu M (2002). Hepatocyte telomere shortening and senescence are general markers of human liver cirrhosis. Faseb j.

[81] Wang Z, Lin H, Hua F, Hu ZW (2013). Repairing DNA damage by XRCC6/KU70 reverses TLR4-deficiency-worsened HCC development via restoring senescence and autophagic flux. Autophagy.

[82] Magdaleno F, Arriazu E, Ruiz de Galarreta M (2016). Cartilage oligomeric matrix protein participates in the pathogenesis of liver fibrosis. J Hepatol.

[83] Arora V, Baweja S, Sarin SK (2020). Letter to the Editors: Relevance of Circulating Extracellular Vesicles Carrying Sphingolipid Cargo in Alcoholic Hepatitis: Need for more validation !. Hepatology.

[84] Rahman MA, Patters BJ, Kodidela S, Kumar S (2020). Extracellular Vesicles: Intercellular Mediators in Alcohol-Induced Pathologies. J Neuroimmune Pharmacol.

[85] Hira E, Ono T, Dhar DK (2005). Overexpression of macrophage migration inhibitory factor induces angiogenesis and deteriorates prognosis after radical resection for hepatocellular carcinoma. Cancer.

[86] Marin V, Poulsen K, Odena G (2017). Hepatocyte-derived macrophage migration inhibitory factor mediates alcohol-induced liver injury in mice and patients. J Hepatol.

[87] Poulsen KL, McMullen MR, Huang E (2019). Novel Role of Macrophage Migration Inhibitory Factor in Upstream Control of the Unfolded Protein Response After Ethanol Feeding in Mice. Alcohol Clin Exp Res.

[88] Costa-Silva B, Aiello NM, Ocean AJ (2015). Pancreatic cancer exosomes initiate pre-metastatic niche formation in the liver. Nat Cell Biol.

[89] Wiśniewski JR, Zougman A, Nagaraj N, Mann M (2009). Universal sample preparation method for proteome analysis. Nat Methods.

[90] Tyanova S, Temu T, Sinitcyn P (2016). The Perseus computational platform for comprehensive analysis of (prote)omics data. Nat Methods.

[91] Huang da W, Sherman BT, Lempicki RA (2009). Systematic and integrative analysis of large gene lists using DAVID bioinformatics resources. Nat Protoc.

[92] Manna SK, Thompson MD, Gonzalez FJ (2015). Application of mass spectrometry-based metabolomics in identification of early noninvasive biomarkers of alcohol-induced liver disease using mouse model. Adv Exp Med Biol.

[93] Zehra M, Curry JC, Pillai SS, Lakhani HV, Edwards CE, Sodhi K (2020). Elucidating Potential Profibrotic Mechanisms of Emerging Biomarkers for Early Prognosis of Hepatic Fibrosis. Int J Mol Sci.

[94] Chen Y, Manna SK, Golla S (2019). Glutathione deficiency-elicited reprogramming of hepatic metabolism protects against alcohol-induced steatosis. Free Radic Biol Med.

[95] Bala S, Petrasek J, Mundkur S (2012). Circulating microRNAs in exosomes indicate hepatocyte injury and inflammation in alcoholic, drug-induced, and inflammatory liver diseases. Hepatology.

[96] Sun N, Lee YT, Zhang RY (2020). Purification of HCC-specific extracellular vesicles on nanosubstrates for early HCC detection by digital scoring. Nat Commun.

[97] Di Tommaso L, Destro A, Fabbris V (2011). Diagnostic accuracy of clathrin heavy chain staining in a marker panel for the diagnosis of small hepatocellular carcinoma. Hepatology.

[98] Stefanini GF, Mazzetti M, Zunarelli P (1989). *In vivo* effect of chronic ethanol abuse on membrane alpha 1-glycoprotein of lymphocytes and immune response to various stimulating agents. Alcohol Clin Exp Res.

[99] Wang WB, Fan JM, Zhang XL, Xu J, Yao W (2009). Serial expression analysis of liver regeneration-related genes in rat regenerating liver. Mol Biotechnol.

[100] Sugihara T, Tanaka S, Braga-Tanaka I 3rd, Murano H, Nakamura-Murano M, Komura JI (2018). Screening of biomarkers for liver adenoma in low-dose-rate γ-ray-irradiated mice. Int J Radiat Biol.

[101] Yamanaka C, Wada H, Eguchi H (2018). Clinical significance of CD13 and epithelial mesenchymal transition (EMT) markers in hepatocellular carcinoma. Jpn J Clin Oncol.

[102] Borowsky SA, Lieberman J, Strome S, Sastre A (1982). Elevation of serum angiotensin-converting enzyme level. Occurrence in alcoholic liver disease. Arch Intern Med.

[103] Leebeek FW, Kluft C, Knot EA, De Maat MP (1989). Histidine-rich glycoprotein is elevated in mild liver cirrhosis and decreased in moderate and severe liver cirrhosis. J Lab Clin Med.

[104] Mani I, Alexopoulou A, Vasilieva L (2019). Human beta-defensin-1 is a highly predictive marker of mortality in patients with acute-on-chronic liver failure. Liver Int.

[105] Leto G, Tumminello FM, Pizzolanti G, Montalto G, Soresi M, Gebbia N (1997). Lysosomal cathepsins B and L and Stefin A blood levels in patients with hepatocellular carcinoma and/or liver cirrhosis: potential clinical implications. Oncology.

[106] Megias MJ, Alba-Araguez F, Luna JD, Vives F, Ramirez-Sanchez M (2015). Serum pyroglutamyl aminopeptidase activity: a promising novel biomarker candidate for liver cirrhosis. Endocr Regul.

[107] Grasselli E, Compalati AD, Voci A (2014). Altered oxidative stress/antioxidant status in blood of alcoholic subjects is associated with alcoholic liver disease. Drug Alcohol Depend.

[108] Lee ES, Kim SH, Kim HJ, Kim KH, Lee BS, Ku BJ (2017). Growth Differentiation Factor 15 Predicts Chronic Liver Disease Severity. Gut Liver.

[109] Chung HK, Kim JT, Kim HW (2017). GDF15 deficiency exacerbates chronic alcohol- and carbon tetrachloride-induced liver injury. Sci Rep.

[110] Lin J, Zhang Y, Wu J (2018). Neuropilin 1 (NRP1) is a novel tumor marker in hepatocellular carcinoma. Clin Chim Acta.

[111] Liu XR, Cai CX, Luo LM (2016). Decreased expression of Sushi Domain Containing 2 correlates to progressive features in patients with hepatocellular carcinoma. Cancer Cell Int.

[112] Cauza E, Maier-Dobersberger T, Polli C, Kaserer K, Kramer L, Ferenci P (1997). Screening for Wilson's disease in patients with liver diseases by serum ceruloplasmin. J Hepatol.

[113] Carvalho JR, Verdelho Machado M (2018). New Insights About Albumin and Liver Disease. Ann Hepatol.

[114] Spinella R, Sawhney R, Jalan R (2016). Albumin in chronic liver disease: structure, functions and therapeutic implications. Hepatol Int.

[115] Kumagi T, Akbar F, Horiike N, Onji M (2001). Increased serum levels of macrophage migration inhibitory factor in alcoholic liver diseases and their expression in liver tissues. Clin Biochem.

[116] Meier EM, Rein-Fischboeck L, Pohl R (2016). Annexin A6 protein is downregulated in human hepatocellular carcinoma. Mol Cell Biochem.

[117] Biguzzi E, Franchi F, Bucciarelli P, Colombo M, Romeo R (2007). Endothelial protein C receptor plasma levels increase in chronic liver disease, while thrombomodulin plasma levels increase only in hepatocellular carcinoma. Thromb Res.

[118] Zheng J, Guo X, Gao X, Liu H, Tu Y, Zhang Y (2014). Overexpression of retinoic acid-induced protein 3 predicts poor prognosis for hepatocellular carcinoma. Clin Transl Oncol.

[119] Li S, Li H, Yang X (2015). Vasorin is a potential serum biomarker and drug target of hepatocarcinoma screened by subtractive-EMSA-SELEX to clinic patient serum. Oncotarget.

[120] Zachou K, Gabeta S, Gatselis NK, Norman GL, Dalekos GN (2017). Cartilage oligomeric matrix protein on the spot for liver fibrosis evaluation: Too early or too late?. Eur J Intern Med.

